# Urbanization and landscape effects on taxonomic and functional wild bee diversity in small towns and rural areas

**DOI:** 10.1186/s12983-025-00594-y

**Published:** 2025-12-12

**Authors:** Weronika Banaszak-Cibicka, Łukasz Dylewski, Joanna Bajon, Joanna T. Białas, Monika Fliszkiewicz

**Affiliations:** Department of Zoology, Poznań University of Life Sciences, Wojska Polskiego 71C, 60-625 Poznań, Poland

**Keywords:** Urbanization, Agricultural intensification, Anthophila, Rural areas, Traits

## Abstract

**Background:**

Planning and managing urban landscapes for greater biodiversity and creating effective conservation strategies requires understanding the relationships between biodiversity and different types of urbanization. Here, we described the variation between small-town and rural areas in two dimensions of biodiversity – taxonomic and functional. We compared community composition and alpha and beta diversity levels of bees between small town and rural sites. We also analyzed the impact of landscape composition on bee communities.

**Results:**

Our results showed that bee abundance, species richness, Shannon–Wiener diversity index, and functional alpha diversity parameters did not differ between small towns and rural areas. Taxonomical overall beta diversity also did not show significant differences between the areas studied. However, we found higher taxonomical turnover and lower taxonomical nestedness for bees in urban areas than in rural areas. Simultaneously, the functional overall beta diversity was higher in rural than urban areas. Moreover, the results showed negative relationships between bees’ abundance and species richness with the density of impervious surface area (ISA) and cropland cover.

**Conclusions:**

Our results show that even very small towns can influence bee communities, causing decrease in dissimilarity at the functional level, and landscape changes such as increased urbanization and crop cover have a significant negative impact on bees.

**Supplementary Information:**

The online version contains supplementary material available at 10.1186/s12983-025-00594-y.

## Background

As pollinators, bees play a vital role not only in nature but also in the economy. Reports of decreased bee numbers and diversity [43] linked to environmental changes caused by human activity have sparked increased interest in bee conservation. Nearly all of the Earth’s ecosystems are now affected by humans, and extensively used semi-natural habitats are increasingly scarce in modern landscapes. Consequently, most species function in strongly transformed or modified environments, meaning that even human-dominated areas play a significant role in biodiversity conservation. Their importance will increase as the human population increases.

Changes in the fauna of urban environments are an extremely relevant and timely topic. Multiple papers have pointed to the negative impact of urbanization on bee diversity (e.g., [13, 15]). However, according to other sources, well-planned and well-managed urban green spaces may be attractive habitats for many bee species (e.g., [24, 60]). For that reason, urban planning and management for greater biodiversity are currently being promoted. However, developing effective conservation strategies requires understanding the relationship between biodiversity and different types of urbanization [52] and unfortunately, most urban bee research is conducted in big cities, while small towns are largely understudied [65]. Consequently, our knowledge of the impact of urbanization on bees and conservation recommendations are based on studies of large cities. However, it is not clear whether the same relationships occur in small towns. Small towns are an essential element of urbanization since they are much more common than large cities, not only in terms of number but also the total area occupied [17, 51].

Urbanization processes are complex and do not always lead to simple species extinction but may cause changes in the structure of communities, which in turn translate into the functioning of ecosystems and ecosystem services [53]. The latest research indicates the validity of a multi-aspect analysis of pollinator insect communities in transformed areas [9, 10]. Such analyses at various levels make it possible to determine the reactions of organisms that are often invisible during simple analyses of local species richness and abundance. Local biodiversity (alpha diversity) is important, but much of the loss of diversity is due to reduced spatiotemporal variability among local assemblages (beta diversity), leading to decrease in dissimilarity [39]. Reduced species exchange and decrease in dissimilarity of pollinator communities in the landscape may be related to environmental disturbances [46]. Ecologists often distinguish two components of beta diversity: species turnover and nestedness [11]. Species turnover and nestedness differ significantly in their biological consequences. Species turnover occurs when species present in one location are absent in the second but are replaced by other species missing in the first [45]. Nestedness occurs when species present in one location are absent from another and are not replaced by additional species, so they constitute a subset of species from richer habitats [62].

Beta diversity, which measures the variation in community composition along environmental gradients, is significant for biodiversity conservation. Research has primarily focused on taxonomic beta diversity; however, to evaluate how bee communities respond to environmental changes, it is vital to consider the diversity of functional traits they hold [18]. Nevertheless, only some studies have considered these two dimensions of beta diversity of wild bees along anthropogenic disturbance gradients [25, 46, 41]. Functional diversity (FD) describes the functional differences between species [64]. Unlike classic diversity indicators, the analysis of functional diversity enables the assessment of the species composition of assemblages in qualitative, not only quantitative, terms. The characteristics of species translate into different sensitivity to anthropogenic disturbances [28]. Different species, therefore, show different responses to the same factors [3] and, perhaps for this reason, also show other population trends over time. To better understand the impact of environmental transformations on groups of organisms, it is necessary to consider not only species diversity but also their functional diversity.

Consequently, we investigated the patterns and determinants of alpha and beta diversity on taxonomic and functional dimensions, as well as how landscape composition (density of impervious surface area (ISA), the cover of road, the cover of forest, the cover of cropland, the cover of built-up area, the cover of waterbodies and length of hydrographic network) influenced bee communities in small-town and rural areas. A study was carried out in the small cities of Western Poland. Its results are an essential first step in better understanding the bee fauna of small towns, especially those in similar climatic and cultural zones. We expected that small towns and rural areas would contain similar taxonomic and functional diversity of bees, as green areas in small towns are relatively close to the areas outside the cities, which facilitates the movement of species between these habitats [8]. Additionally, both small towns and rural areas are transformed habitats, where human activities affect pollinator communities, leading to a reduction in biodiversity [35]. We also predicted the impact of landscape composition on bee communities. Specifically, we hypothesized that increased urbanization and cropland cover would negatively impact bee abundance and species richness, as both urban and agricultural intensification are recognized as significant factors contributing to the decline in bee diversity [20, 61]. In contrast, we expected woodland cover to have a positive effect on bee assemblages. Growing evidence indicates that forests can support wild bee communities in temperate regions [1, 63], and higher woodland cover has been repeatedly associated with increased wild be abundance and/or diversity (e.g., [2, 54, 57]).

## Methods

### Study area

**Fig. 1:**
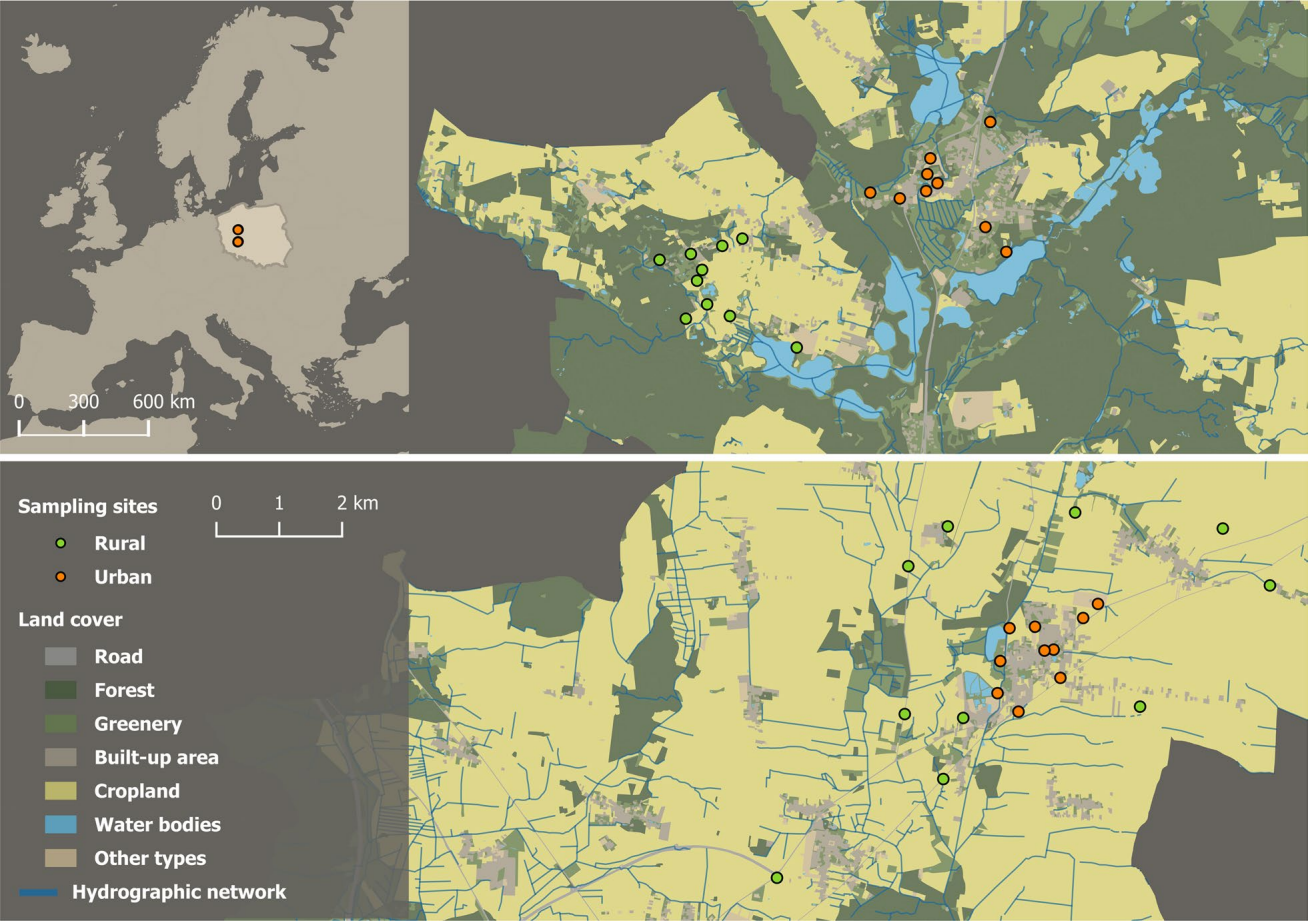
Maps show the location of urban and rural study sites within Europe (top left) and within their respective communes (top right and bottom), with land cover types visualized on the latter. Land cover data are based on the BDOT10k dataset obtained from the national database of topographic objects, provided by the head office of geodesy and cartography, Poland

The research was carried out in two small towns, Miejska Górka and Skoki, and their surrounding areas, all situated in the Greater Poland Voivodeship in western Poland (Fig. 1). Miejska Górka has a population of 3,186 inhabitants, whereas Skoki boasts a population of 4,505. The samples were taken in two types of habitats: urban and rural. The areas were determined on the basis of the distance from the town center and their characteristics. Urban sites were situated within the administrative boundaries of the studied cities and included home gardens surrounded by single- and multi-family houses, grasslands and wastelands close to houses, housing estates, or urban infrastructure. Rural sites, located outside city limits, consisted primarily of farmland area with minimal built infrastructure where sampling were carried in field margins, semi-natural grasslands, and abandoned fields. In order to describe the degree of area modification resulting from human activity, the area with man-made structures, the cover of forest, the cover of cropland, the cover of waterbodies, and the length of the hydrographic network were calculated at each research site. We intend to quantify habitat composition in a 250-m area from the central point of each site as a close neighborhood of nesting and foraging resources within a few hundred meters, which is crucial to maintaining populations of bees [68]. The buffers of urban areas did not cover rural areas (outside city boundaries), and vice versa. Urban areas were characterized by a significantly higher percentage of impervious surface area (ISA), which includes roadways, building coverage, and paved areas and serves as a robust proxy for urbanization intensity. This variable effectively captures the influence of urban development and associated environmental changes (e.g., habitat alteration, human activity, microclimatic variation). In contrast, rural areas featured a significantly greater proportion of arable land compared to urban environments (Table 1 summarizes the differences in environmental variables between rural and urban habitats). A total of 39 research sites were designated (19 urban – 10 in Miejska Górka, 9 in Skoki and 20 rural – 10 in Miejska Górka, 10 in Skoki). Individual sites were spaced at least 500 m apart. These buffers were established to accommodate the anticipated foraging range of most wild bees [68].

**Table 1:** Difference in environmental variables between rural and urban habitats

	Rural		Urban					
	Mean	SE	Mean	SE	F	df	*p*	Effect size
ISA	**4.35**	**2.71**	**22.46**	**2.78**	**21.78**	**1,37**	** < 0.0001**	**2.066**
road	**0.01**	**0.01**	**0.03**	**0.01**	**7.43**	**1,37**	**0.009**	**0.459**
watercourse	39.4	25.9	97.6	64.8	0.94	1,37	0.336	0.431
forest	0.12	0.03	0.09	0.03	0.18	1,37	0.683	0.074
greenery	**0.12**	**0.03**	**0.26**	**0.19**	**7.92**	**1,37**	**0.007**	**0.480**
cropland	**0.62**	**0.06**	**0.21**	**0.06**	**21.28**	**1,37**	** < 0.0001**	**0.721**
built-up area	**0.06**	**0.03**	**0.25**	**0.03**	**19.25**	**1,37**	** < 0.0001**	**0.828**
water bodies	0.03	0.01	0.05	0.01	0.74	1,37	0.395	0.146

ISA, mean density of impervious surface area (%); road, cover of road (proportion); water courses, sum of water courses lengths (in meter logarithm scale); forest, cover of deciduous, conifer and mixed forest (proportion); greenery, cover of grassland (proportion); cropland, cover of arable land (proportion); built-up area, cover of buildings and carped area (proportion); water bodies, cover of water bodies (proportion). Significant results (*p* < 0.05) are in bold

### Data collection

The field survey was conducted from May to the end of September 2021. Bees were collected using an entomological net. The bumblebees and species easy to distinguish were identified on-site in vivo. The rest were collected for further identification in the laboratory. Whereas all bees were identified to the species level, the subgenus *Bombus *sensu stricto, encompassing four species in Poland (*Bombus terrestris*, *B. lucorum*, *B. cryptarum*, and *B. magnus*), posed challenges for field identification, particularly in distinguishing the workers. Due to the complexity of distinguishing these species in the field, they were classified as *Bombus* s. str.

Bees were collected at all sampling sites along transects. Each transect was 200 m long and 1 m wide. The direct search and collecting bees was carried out in 30 min on each site. All visits were conducted between 8 am and 6 pm in weather conditions favorable for bee activity [10]. Catches were carried out on sunny and warm days, without rainfall, with moderate wind. Research sites were visited at random order so that samples from each site were taken at different times of the day. Each site was visited six times. Information on wild bee species traits was obtained from several published trait databases. We used mean body length, nesting place (cavity, soil, or hive), social behavior (solitary, eusocial, or cleptoparasitic), floral specificity (oligolectic—single plant species or genus, polylectic—many plant species), flight beginning period (March/April, May/June, July/August), end of flight period (May/June, July/August, September/October) and flight period (months of activity) extracted from following literature used also for species identification [5–7, 40, 42, 48, 49].

### Spatial analyses

We performed spatial analysis within the buffers of 250 m around each sampling point. We calculated the mean value of the density of impervious surface area (ISA), the cover of roads, the cover of the forest, the cover of cropland, the cover of built-up area, the cover of waterbodies, and the length of the hydrographic network. To compute ISA density, we used the Copernicus Land Monitoring Service Imperviousness Degree 2021 dataset, which provides the percentage of sealed surfaces (0–100%) at ~ 10 m spatial resolution. The data are based on satellite imagery from 2021. The ISA density represents the proportion of constructed surfaces, including buildings and infrastructure networks, within a given area. The dataset is publicly available at https://land.copernicus.eu. The vector maps depicting land cover and the hydrographic network at a scale of 1:10,000 were sourced from the National Database of Topographic Objects through the Head Office of Geodesy and Cartography. The hydrographic network data encompassed the lengths of watercourses, incorporating small drainage ditches, canals and rivers. All spatial analyses were conducted using QGIS Desktop 3.16.3.

### Statistical analyses

We analyzed two aspects of bee diversity—taxonomic and functional, at two levels – alpha (within-site) and beta (among sites). We calculated taxonomic alpha diversity using species richness and Shannon’s diversity index. We calculated functional diversity by functional richness (FRic), expressing the quantity of bee functional types present in a community, functional dispersion (FDis), expressing the size of community species traits hypervolume within the functional trait space, functional divergence (FDiv), expressing on how large the average distance of each species to the center of gravity (center-space) of the trait space, and functional evenness (FEve), informing about on the degree of evenness of the distribution of biomass in a niche space [27, 33, 44]. Moreover, we calculated community-weighted mean (CWMs) values of functional bee traits for each site to account for species abundance.

We calculated beta diversity indices using Jaccard’s dissimilarity index, as this metric was the most frequently used in previous studies on biotic homogenization [39]. Taxonomic beta diversity was calculated using a species presence-absence matrix, functional diversity – using the volume of convex hull intersections in a multidimensional functional space (extracted from principal coordinates analysis from species traits of a Gower dissimilarity matrix) whereas phylogenetic diversity – using a matrix of phylogenetic distances. These indices were calculated using the betapart package [12, 47]. For each beta diversity index, we calculated the overall value, nestedness, and turnover [11]. This allowed us to explain the importance of nestedness (presence of core species) and turnover (species replacement) in shaping dissimilarities among particular habitat types and studies.

We checked for potential spatial autocorrelation using Moran’s I test. The results indicated that spatial autocorrelation in the residuals was not significant (*p*-value < 0.05) (Supplementary Materials, Table S1).

We used mixed models with beta distributions to assess differences between urban and rural habitats in beta-diversity indices. We applied a linear transformation to eliminate zeroes and ones prior to conducting mixed models with beta distribution [55]. We used sampling site ID as a random factor in all constructed models.

To compare the composition of wild bee species between urban and rural habitats, we used non-metric multidimensional scaling (NMDS) with the metaMDS function based on Bray–Curtis distance and three dimensions. The significance of fitted vectors was assessed using permutation tests with 999 random permutations of the data. The multivariate permutation analysis of variance PERMANOVA was used to check differences between centroids and the dispersion of groups representing urban and rural habitats using the adonis function implemented in the vegan package [38].

To identify environmental landscape variables and habitat type affecting wild bee abundance, species richness, and alpha taxonomical and functional diversity, we used a generalized linear mixed model with a restricted maximum-likelihood (REML) estimator implemented and sampling site ID as a random factor. We used negative binomial distributions of abundance and species richness and Gaussian distributions for Shannon diversity index. For the functional parameters, we used beta distributions.

As explanatory variables, we used habitat type (rural vs. urban), the density of impervious surface area (ISA, as a metric of urbanization, allows us to determine the urban–rural gradient in our study), the cover of grassland, the cover of road, the cover of forest, the cover of cropland, the cover of built-up area, the cover of waterbodies and length of the hydrographic network (log_10_(+ 1) transform) on each model. We also included interactions between habitat type and environmental variables. Next, we used Akaike’s information criterion for small sample sizes (AICc) to compare the models with interactions with models without interaction terms to select the final models (with the lowest AICc). To avoid multicollinearity, we excluded the cover of grasslands and the cover of built-up areas from all models (Supplementary Materials, Fig. S1). Multicollinearity in the remaining explanatory variables in both models was not excessive (VIF < 2, according to [69]). Moreover, we calculated the Cohen f effect size for each models.

Statistical analysis was conducted in R 4.3.1 (R Core Developmental R Core Team 2023) and CANOCO 5 [58, 59]. GLMMs were carried out using the lme4 (Gaussian and negative binomial distribution) and glmmTMB (beta distribution) packages [14, 16], the effect size for each models were calculated in sjstats package (Lüdecke, 2020), the NMDS and PERMANOVA were carried out in the vegan package [38], and the data visualization using the ggplot2 package [66].

## Results

### Bee community composition

In total 3451 wild bee specimens of 107 species were recorded, 1671 individuals were found on sites located in small towns, and 1783 on rural sites. A total of 81 species of bees were found in urban and 86 in rural areas.

**Fig. 2:**
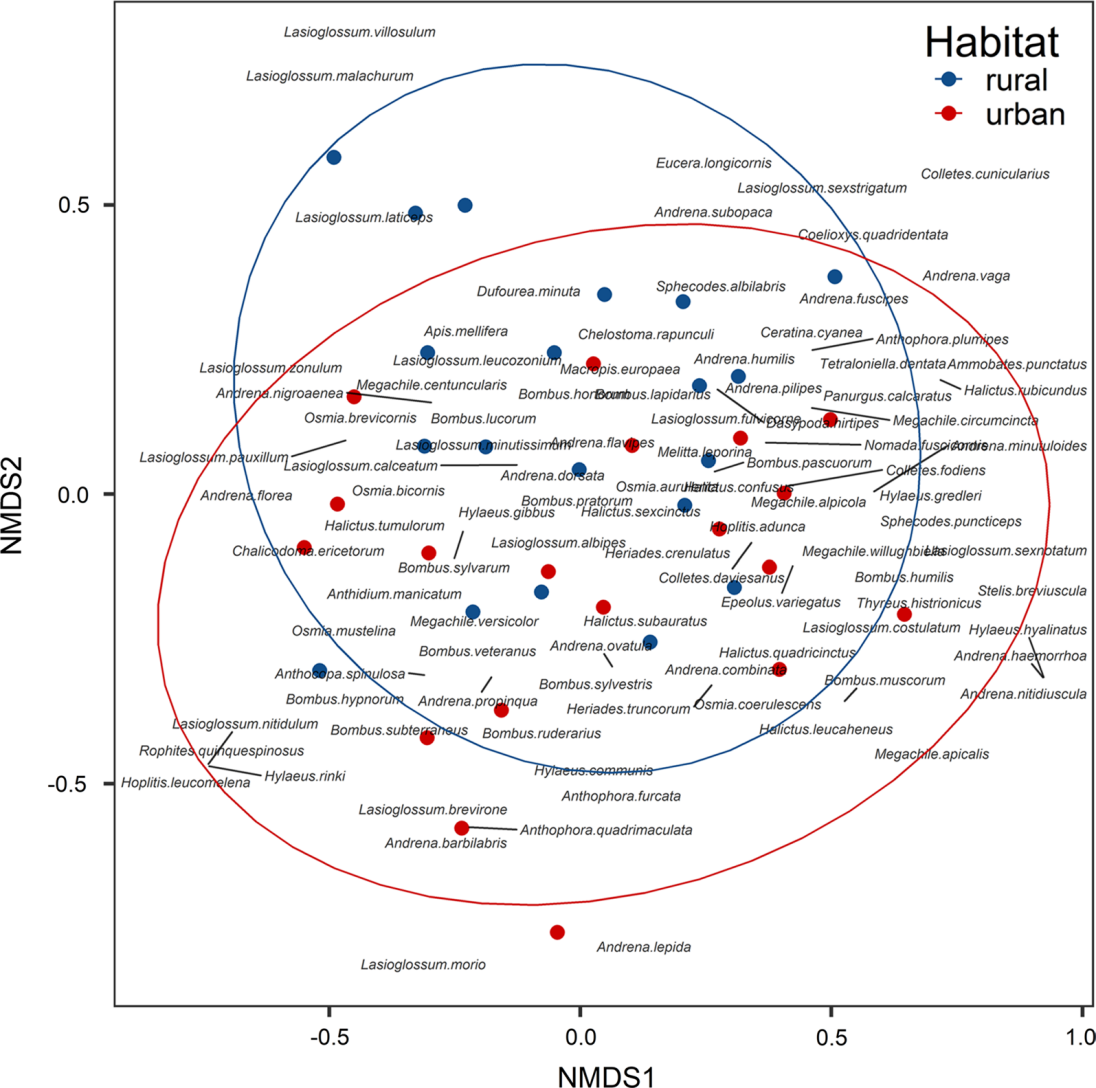
The non-metric multidimensional scaling (NDMS) analysis of bee species composition on the study plots. The dots represent each site. The stress parameter was 0.165

The most abundant bee species were *Apis mellifera* (Linnaeus, 1758) contributed nearly 19% of visits, *Bombus lapidarius* (Linnaeus, 1758) 18%, *Bombus s. str*. 14%, *Bombus pascuorum* (Scopoli, 1763) 10%, *Bombus hypnorum* (Linnaeus, 1758) 6%, and *Colletes fodiens* (Fourcroy, 1785) 3%. Apart from the common species, there were 8 species from Polish Red List (Banaszak 2002). They were classified as Vulnerable (VU): *Andrena florea* Fabricius, 1793, *Andrena lepida* Schneck, 1861, *Bombus humilis* Illiger, 1806, *Bombus veteranus* (Fabricius, 1793), *Hylaeus rinki* (Gorski, 1852), and classified as Data deficient (DD): *Dufourea minuta* Lepeletier, 1845, *Hylaeus gredleri* Förster, 1871, and *Thyreus histrionicus* (Illiger, 1806). Most of the these species (5) occurred in higher numbers or exclusively in urban areas. A large proportion of all species was observed in both types of habitats, however, 22 species of bees occurred only in urban areas, and 27 only in rural areas. All species that have been observed belong to native species. The NMDS analysis has showed non-significant differences between urban and rural areas in bee communities (F_1,37_ = 1.37, *p* = 178, R^2^ = 0.03, Fig. 2).

### Taxonomical and functional beta diversity of wild bees

**Fig. 3:**
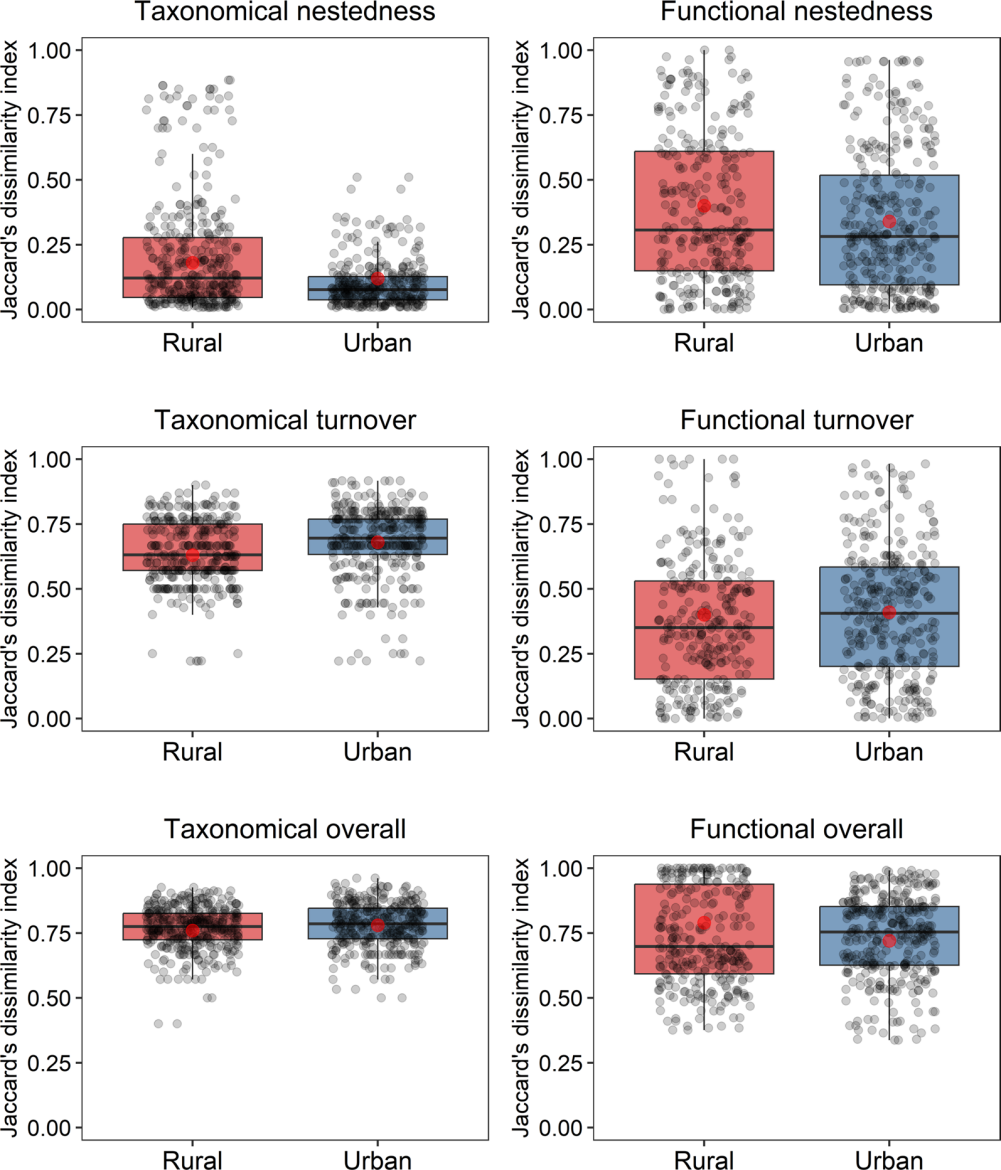
Boxplot of taxonomic and functional turnover, nestedness, and overall beta diversity of bees in rural and urban areas. Red dots indicate mean values, and each point represents a single observation

Taxonomical overall beta diversity did not differ between studied areas. However, we found higher taxonomical turnover for bees (Wald = 7.49, *p* = 0.006) in urban areas as compared to rural and lower taxonomical nestedness (Wald = 8.62, *p* = 0.003) in urban when compared with rural areas (Fig. 3). The functional overall beta diversity was higher in rural than in urban areas (Wald = 4.08, *p* = 0.043), whereas there were no significant differences in functional nestedness and turnover between the habitats (*p* > 0.05, Table 2).

**Table 2:** Difference in taxonomic and functional beta diversity of wild bees between rural and urban habitats

	Rural		Urban					Effect size Cohens f
	Mean	SE	Mean	SE	Wald	df	*p*	
Taxonomical overall	0.76	0.01	0.78	0.01	2.35	1	0.125	0.048
Taxonomical nestedness	**0.18**	**0.01**	**0.12**	**0.01**	**8.62**	**1**	**0.003**	**0.124**
Taxonomical turnover	**0.63**	**0.01**	**0.68**	**0.01**	**7.49**	**1**	**0.006**	**0.102**
Functional overall	**0.79**	**0.02**	**0.72**	**0.02**	**4.08**	**1**	**0.043**	**0.005**
Functional nestedness	0.40	0.02	0.34	0.02	3.08	1	0.078	0.056
Functional turnover	0.40	0.03	0.41	0.02	0.01	1	0.927	0.034

Significant results (*p* < 0.05) are in bold

### Environmental landscape variables influence on wild bee diversity in urban–rural gradient

Our results showed that bee abundance (z = − 0.87, *p* = 0.406), species richness (z = − 0.33, *p* = 0.744) and Shannon diversity index (z = − 0.88, *p* = 0.383) did not differ between small towns and rural areas (Supplementary Materials, Fig. S2). Similarly, the functional alpha diversity parameters were non-significant (*p* > 0.05, Table 3).

**Table 3:** The results of the generalized linear models describing the relationship between the abundance, alpha taxonomical and functional diversity of bees and environmental variables

	Estimate	SE	z/t	*p*	Cohens f
*Abundance*	**R**^**2**^_**m**_** = 0.39**	**R**^**2**^_**c**_** = 0.98**			
Habitat:urban	− 0.339	0.43	− 0.87	0.406	0.322
ISA	− 0.020	0.02	− 1.37	0.065	0.256
water courses	− 0.114	0.12	− 1.93	0.302	0.218
road	0.329	4.52	0.07	0.946	0.387
forest	− 0.309	1.19	− 0.25	0.617	1.478
cropland	− **2.459**	**0.75**	− **3.26**	**0.001**	**1.165**
water bodies	− 1.424	1.94	− 0.73	0.358	0.410
*Species richness*	**R**^**2**^_**m**_** = 0.43**	**R**^**2**^_**c**_** = 0.59**			
Habitat:urban	− 0.054	0.17	− 0.33	0.744	0.123
ISA	− **0.013**	**0.01**	− **2.26**	**0.023**	**0.298**
water courses	− 0.052	0.05	− 1.04	0.299	0.090
road	1.752	0.98	1.78	0.074	0.026
forest	0.161	0.40	0.40	0.690	0.704
cropland	− **0.966**	**0.26**	− **3.74**	** < 0.0001**	**0.548**
water bodies	− 0.511	0.60	− 0.85	0.395	0.112
*Alpha diversity*	**R**^**2**^_**m**_** = 0.25**	**R**^**2**^_**c**_** = 0.90**			
Habitat:urban	− 0.224	0.25	− 0.88	0.383	0.428
ISA	− 0.005	0.01	− 0.60	0.554	0.290
water courses	− 0.047	0.07	− 0.66	0.517	0.317
road	4.426	3.06	1.45	0.158	0.700
forest	0.324	0.73	0.44	0.660	0.215
cropland	− 0.43	0.46	− 1.62	0.116	0.781
water bodies	0.898	1.23	0.73	0.471	0.353
*Functional dispersion*	**R**^**2**^_**m**_** = 0.15**	**R**^**2**^_**c**_** = 0.88**			
Habitat:urban	− 0.044	0.05	− 0.80	0431	0.386
ISA	0.001	0.01	0.18	0.859	0.086
water courses	− 0.007	0.02	− 0.45	0.659	0.216
road	0.749	0.67	1.12	0.271	0.541
forest	0.090	0.16	0.57	0.576	0.274
cropland	− 0.102	0.11	− 1.02	0.316	0.493
water bodies	0.111	0.27	0.41	0.681	0.200
*Functional divergence*	**R**^**2**^_**m**_** = 0.18**	**R**^**2**^_**c**_** = 0.88**			
Habitat:urban	0.011	0.07	0.15	0.880	0.073
ISA	0.001	0.00	0.37	0.713	0.179
water courses	− 0.016	0.02	− 0.79	0.433	0.384
road	0.029	0.87	0.34	0.973	0.017
forest	− 0.062	0.21	− 0.30	0.766	0.145
cropland	− 0.098	0.13	− 0.75	0.462	0.361
water bodies	0.453	0.35	1.29	0.205	0.626
*Functional richness*	**R**^**2**^_**m**_** = 0.15**	**R**^**2**^_**c**_** = 0.88**			
Habitat:urban	− 0.017	0.18	− 0.09	0.923	0.047
ISA	− 0.005	0.01	0.76	0.451	0.369
water courses	− 0.063	0.05	− 1.22	0.229	0.593
road	2.544	2.19	1.15	0.256	0.560
forest	− 0.225	0.52	− 0.43	0.671	0.207
cropland	− 0.349	0.33	− 1.06	0.299	0.511
water bodies	0.932	0.88	1.05	0.300	0.510
*Functional evenness*	**R**^**2**^_**m**_** = 0.15**	**R**^**2**^_**c**_** = 0.88**			
Habitat:urban	− 0.082	0.08	− 1.00	0.324	0.485
ISA	0.003	0.01	1.18	0.248	0.569
water courses	0.019	0.02	0.83	0.415	0.400
road	1.840	0.98	1.87	0.071	0.906
forest	0.102	0.24	0.44	0.665	0.211
cropland	− 0.005	0.15	− 0.04	0.970	0.018
water bodies	0.507	0.39	1.29	0.208	0.621

SE, standard error; z/t, test statistic (for all variables z, except alpha diversity assessed using LMM assuming normal distributions); effect size, Cohen’s f statistic; R^2^_m_, marginal coefficient of determination; R^2^_c_– conditional coefficient of determination; Habitat, type of habitat; rural versus urban, ISA, mean density of impervious surface area (%), water courses, sum of water courses lengths (in logarithm scale); road, cover of road (proportion), forest cover of deciduous; conifer and mixed forest (proportion), cropland, cover of arable land (proportion), water bodies, cover of water bodies (proportion). Significant results (*p* < 0.05) are in bold

**Fig. 4:**
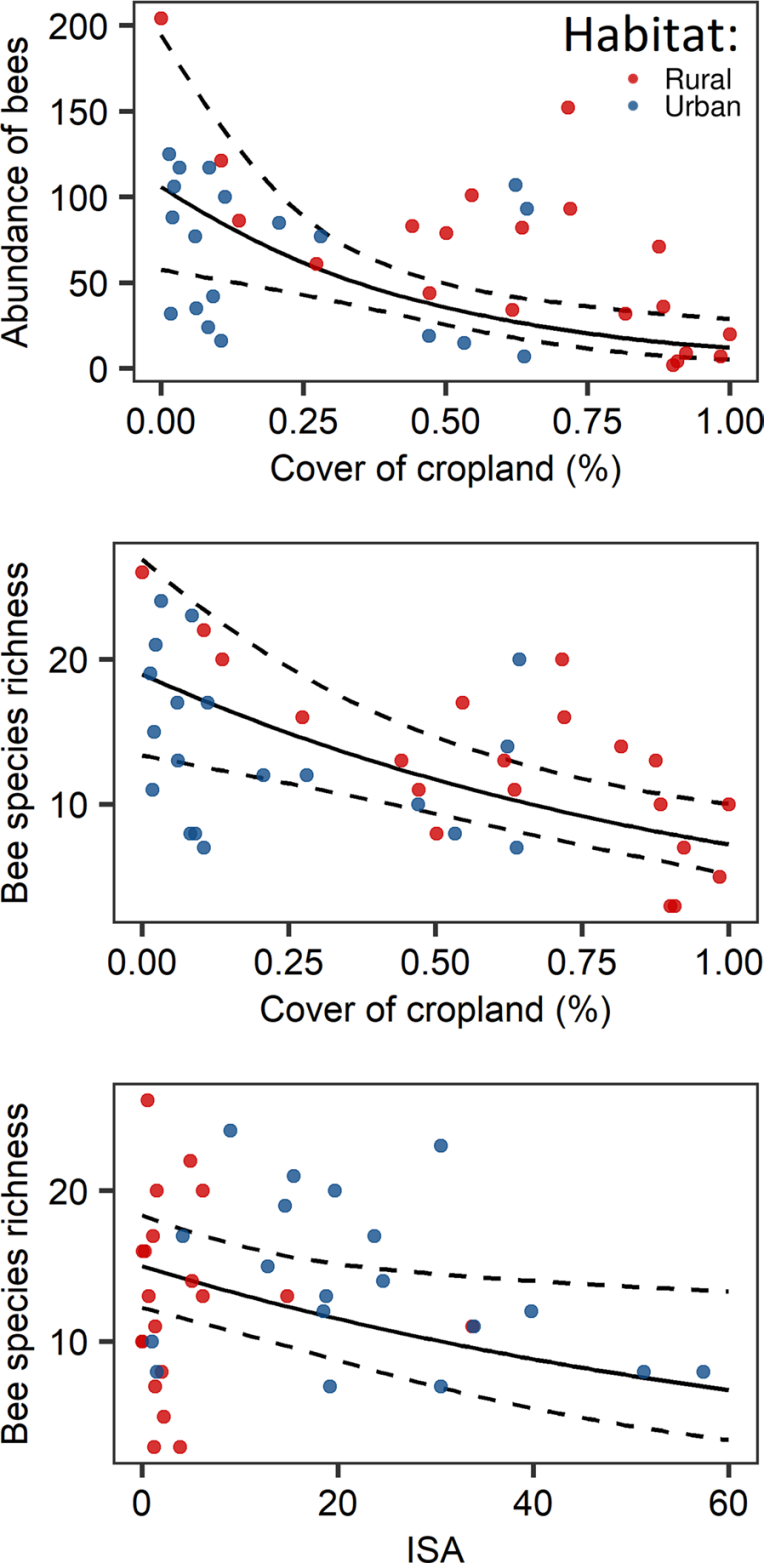
The relationship between percent cover of cropland on abundance and species richness and impervious surface area (ISA) on species richness of bees. Each point represents one sampling event, the black line represents the best-fit generalized linear mixed model, and the dotted lines represent a 95% confidence interval

We assessed negative relationships between species richness of bees with ISA (richness: z = − 2.26, *p* = 0.023) and abundance and species richness with the cover of cropland (abundance: z = − 3.26, *p* = 0.001, richness: z = − 3.74, *p* < 0.0001) (Fig. 4, Tab. 3). For the alpha and functional diversity we did not find any significant relationship with environmental variables (*p* > 0.05, Table 3).

## Discussion

Our results showed that bee abundance, species richness, Shannon diversity index, and functional alpha diversity parameters did not differ in small towns and rural areas. Taxonomical overall beta diversity also did not show significant differences between the studied areas. However, we found higher taxonomical turnover and lower taxonomical nestedness for bees in urban areas than rural areas. Simultaneously, the functional overall beta diversity was lower in urban than rural areas. Moreover, the results showed negative relationships between bee species richness and the density of impervious surface area (ISA) and bee abundance and species richness and cropland cover. As the research was conducted only in cities in Western Poland, they have spatial limitations and generalization of these results should be done with cognizance of the local nature of the study. However, the results can be used as baseline data for future efforts that may be conducted to further evaluation of small-town areas for bees. Extending the research to other countries might be interesting in order to understand whether the described relationships are geographically specific.

The 107 bee species (81 in urban areas and 86 in rural areas) observed during the study comprised over 30% of the species recorded from the region (the Wielkopolska-Kujavian Lowland, Banaszak 2010). Although it should be noted that in the long-term study in the Wielkopolska-Kujawy Lowland, bees were examined not only in rural areas and urban settings, but also in natural habitats (Wielkopolski National Park and natural reserves). Our findings confirmed that small towns have the potential to support diverse pollinator assemblages (25% of regional fauna), consisting not only of common but also threatened species. Small towns were favorable habitats in this respect, mainly since most of red-listed species occurred in higher numbers or exclusively in urban areas. Moreover, all observed species were native. This is a relevant pattern from the conservation point of view of this vital group of pollinating insects. Many publications indicate a lower alpha diversity of bees in urban environments than rural areas (e.g., [13, 19]). Still, other studies also show no differences or opposite tendencies (see [4, 24]). Nevertheless, taxonomic alpha diversity analyses may overlook the effects of land use change on assemblages dissimilarity [56], whereas beta diversity analyses effectively capture potential compositional variations between assemblages.

Although overall taxonomical beta diversity in our study did not differ between small towns and rural areas, taxonomical turnover was significantly higher in urban areas compared to rural areas. Higher taxonomical turnover of the bee community in urban areas suggests that urban environments foster greater species replacement, potentially due to increased habitat heterogeneity in cities. Conversely, we observed higher nestedness in rural areas. This means that some species present in one location were absent in others and were not replaced by additional species [56]. This pattern indicates that in rural areas, poorer sites (in terms of species richness and abundance) constituted a subset of the more diverse ones [62].

Urbanization has previously been linked to a decrease in dissimilarity among communities which may pose a threat to biodiversity (e.g., [23]). Whereas much attention has been focused on taxonomic dissimilarity, the functional aspects of biotic dissimilarity in cities are much less understood [30]. Our study did not show the decrease in taxonomic dissimilarity in urban as compared with rural areas. However, our results suggest that small-town areas cause the decrease in functional dissimilarity. This indicates that species in urban areas tend to share more similar traits or ecological roles, likely due to selective pressures favoring species that can thrive in urban habitats (e.g., generalists, species tolerant of human disturbance). Loss of functional beta diversity can occur independently from species taxonomic dissimilarity. Species diversity can remain relatively unchanged whereas functional diversity decreases [21, 50]. Urban areas can support high taxonomic diversity [23, 26] but lower functional diversity [29, 36] than adjacent areas. Changes in habitat may not be followed by reduced species diversity or abundance, which could lead to the misconception that environmental changes do not affect the studied communities in any way. In these cases, an in-depth analysis of functional traits may point to the contrary. Our results show that changes in community composition can be observed at the functional level, even without species‐level decrease in dissimilarity. Small-town areas support species-rich communities yet, they tend to be functionally more similar due to selective pressures favoring species adapted to urban settings. The higher taxonomical turnover could indicate ongoing species replacement, which may temporarily mask the longer-term trend toward decrease in dissimilarity. This distinction between functional and taxonomical aspects is critical for interpreting the results. Environmental filtering apparently determines the decreased dissimilarity of functional roles in urban bee species. More stressful environments restrict certain combinations of traits, excluding species with maladaptive traits in a specific ecosystem. Conversely, weaker environmental filtering in rural areas allows for greater functional diversity. The lower functional diversity indicates a simplification of the whole ecosystem and has been associated with decreased ecosystem resilience [39]. Some limitations of our study arise from the variation in distances between sampling sites. Because these distances were not standardized, differences in spatial proximity may have influenced patterns of community similarity, turnover, and nestedness. Bees and other pollinators have species-specific dispersal abilities, and shorter distances between sites could lead to higher overlap in species composition. Therefore, some of the observed differences between rural and urban areas may partly reflect spatial structure rather than purely habitat effects*.* Future studies could address this issue by standardizing inter-site distances. Although transects were positioned within defined sampling areas, the spatial structure of our rural–small city gradient did not allow us to quantify or control for edge effects systematically. Nonetheless, because impervious surface density was explicitly incorporated as a measure of urbanization intensity, we consider any potential edge effects to be minimal and unlikely to have biased comparisons between rural and urban sites.

Our results demonstrate that both increased urbanization and agricultural intensification have a negative impact on bee species’ abundance and richness. The growing density of the impervious surface area (ISA) and cropland area results in an effective loss of habitats that provide suitable food resources and nesting sites for bees. Urbanization and modern agriculture have drastically changed landscapes and can have detrimental impacts on pollinator communities. Our results are consistent with the findings of previous studies. It is found that increasing impervious surface area decreases the richness and abundance of bees [13, 22]. Also, high cropland cover is associated with bee declines [32, 37]. As impervious surface and cropland cover area increases, refuge habitats become scarce and are more dispersed over a landscape. Habitat loss is generally considered the most crucial factor causing bee declines [67]. Thus, the hostility of rural and urban landscapes appears to be mainly related to the need for more nesting sites and floral resources. An adequate amount of green habitats and their appropriate management contribute to improving the quality of small-town and rural areas by providing nesting sites and continuous, abundant, and diverse floral resources, and thereby their ability to promote pollinators. Our study contributes to evidence that anthropogenic land use drives changes in bee species richness and abundance. However, we did not observe any effect of woodland cover on bee communities. The absence of a clear relationship may stem from specific characteristics of the forests themselves. Indeed, forest composition appears to be critical: bee richness has been shown to increase with the diversity of insect-pollinated trees but decrease with a higher proportion of coniferous trees (Traylor et al., 2024). Therefore, not all forested habitats provide equal benefits for wild bees, and local forest traits may largely determine their value for bee communities. Nevertheless, evidence suggests that proximity to semi-natural habitats and habitat corridors can ameliorate potential adverse effects [34]. Proper management of human-dominated areas is essential for ensuring the well-being and sustainability of pollinator populations in urban and rural environments. Previous studies on bees in urban and rural environments have stressed the need to increase landscape heterogeneity and connectivity to maintain diverse pollinator communities (e.g., [8]). Thus, local management can secure populations with opportunities to benefit rural and urban landscapes for the benefit of biodiversity and society.

## Management implications

In conclusion, our study provides valuable insights into the diversity of bee species within small towns. Bee richness, abundance, alpha and beta diversity on the taxonomical level of bee assemblages in small-town and rural areas did not differ. Thus, small towns can harbor taxonomically diverse pollinator communities, including species of conservation concern. Simultaneously, differentiating the components of beta diversity (turnover and nestedness) results in different implications for conservation strategies. As taxonomic beta diversity was mainly composed of the turnover component, proving species replacements between sites in the small-town areas, efforts targeted at bee conservation should not be concentrated in one city area. Instead, protection across various urban habitats is a more appropriate strategy for bee diversity conservation in small towns. Our findings also revealed a trend of decreased dissimilarity in functional diversity in small towns. These results show that even very small towns can influence bee communities and landscape changes such as increased urbanization and crop cover have a significant negative impact on bees. Furthermore, our studies underscore the importance of analyzing both taxonomic and functional diversity to improve our understanding of the ecological impacts of environmental changes.

This study serves as an essential initial step in characterizing bee diversity within small towns and their rural surroundings, emphasizing the need for further research. By expanding our knowledge in this field, we can develop targeted approaches that safeguard pollinator populations. More studies of small cities are needed to assess their impact on biodiversity better. This knowledge can then be applied to better planning for urban wildlife.

## Supplementary Information

Supplementary Material 1.

## Data Availability

The datasets analyzed during the current study are available from the corresponding author on reasonable request.
