# Supplementary material for: Urbanization and landscape effects on taxonomic and functional wild bee diversity in small towns and rural areas
Source: Front Zool. 2025 Dec 12;23:2. doi: 10.1186/s12983-025-00594-y (PMC12805769; doi:10.1186/s12983-025-00594-y)

**Supplementary Materials**

**Urbanization and landscape effects on taxonomic and functional wild bee diversity in small towns and rural areas**

Weronika Banaszak-Cibicka, Łukasz Dylewski, Joanna Bajon, Joanna T. Białas, Monika Fliszkiewicz

Department of Zoology, Poznań University of Life Sciences, Wojska Polskiego 71C, 60-625 Poznań, Poland

Fig. S1 Correlation plot of explanatory variables.

Tab. S1 Result of Moran I statistics.

Fig. S2. Boxplot of abundance, species richness and Shannon-Wiener diversity index for bees, in small towns and rural areas.

Figure S1. Correlation plot of explanatory variables.


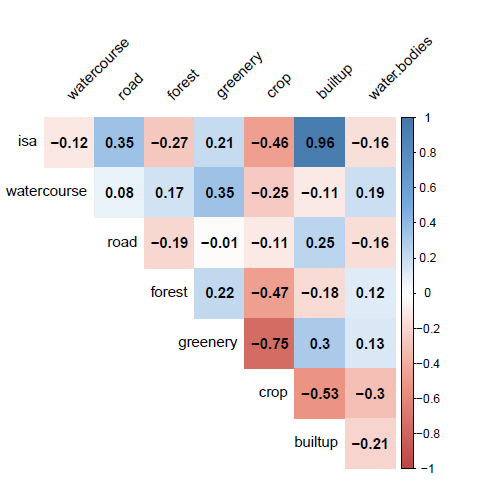


Table S1. Result of Moran I statistics.

|  | Moran I | p-value |
| --- | --- | --- |
| Bee abundance | 0.050 | 0.115 |
| Bee species richness | -0.054 | 0.661 |
| Alpha diversity (Shannon-Wiener) | -0.001 | 0.358 |
| Functional Dispersion | 0.0026 | 0.329 |
| Functional Richness | -0.024 | 0.543 |
| Functional Diversity | -0.078 | 0.779 |
| Functional Evenness | -0.033 | 0.545 |

Figure S2. Boxplot of abundance, species richness and Shannon-Wiener diversity index for bees, in small towns and rural areas.


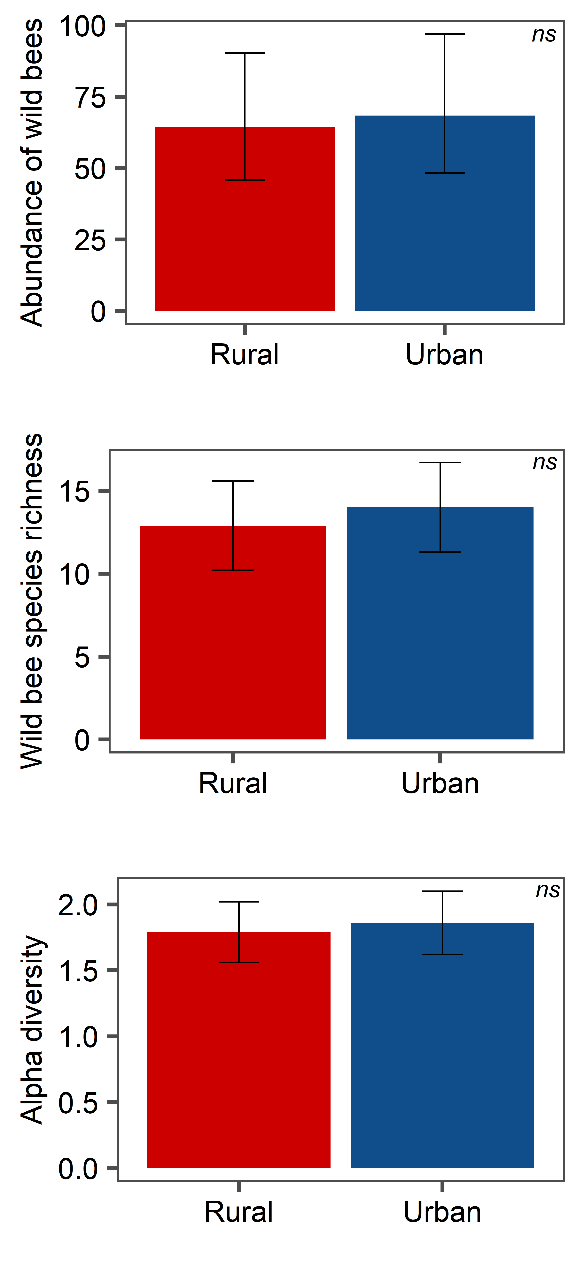

Supplement: Supplementary file 1 — Supplementary Material 1. [file 12983_2025_594_MOESM1_ESM.docx]
